# Positive Association of Serum Vitamin B6 Levels with Intrapulmonary Lymph Node and/or Localized Pleural Metastases in Non-Small Cell Lung Cancer: A Retrospective Study

**DOI:** 10.3390/nu15102340

**Published:** 2023-05-17

**Authors:** Lu Liu, Hang Yu, Jingmin Bai, Qing Xu, Yong Zhang, Xinsheng Zhang, Zhimeng Yu, Yinghua Liu

**Affiliations:** 1 Department of Nutrition, The First Medical Center of Chinese PLA General Hospital, Beijing 100853, China; deer_3016@163.com (L.L.); aqing_930@163.com (Q.X.); zhangyong301@126.com (Y.Z.); zhangxinsheng198431@126.com (X.Z.); yzm18701180993@163.com (Z.Y.); 2 Department of Respiratory and Critical Medicine, Medical School of Chinese People’s Liberation Army, Beijing 100853, China; yuhang_301@126.com; 3 Department of Radiotherapy, The First Medical Center of Chinese PLA General Hospital, Beijing 100853, China; dr_bai@foxmail.com; 4 National Clinical Research Center for Geriatric Diseases, Chinese PLA General Hospital, Beijing 100853, China

**Keywords:** vitamin B_6_, non-small cell lung cancer (NSCLC), TNM stage, lymph node metastasis, localized pleural metastasis

## Abstract

The relationship between vitamin B levels and the development and progression of lung cancer remains inconclusive. We aimed to investigate the relationship between B vitamins and intrapulmonary lymph nodes as well as localized pleural metastases in patients with non-small cell lung cancer (NSCLC). This was a retrospective study including patients who underwent lung surgery for suspected NSCLC at our institution from January 2016 to December 2018. Logistic regression models were used to evaluate the associations between serum B vitamin levels and intrapulmonary lymph node and/or localized pleural metastases. Stratified analysis was performed according to different clinical characteristics and tumor types. A total of 1498 patients were included in the analyses. Serum vitamin B_6_ levels showed a positive association with intrapulmonary metastasis in a multivariate logistic regression (odds ratio (OR) of 1.016, 95% confidence interval (CI) of 1.002–1.031, *p* = 0.021). After multivariable adjustment, we found a high risk of intrapulmonary metastasis in patients with high serum vitamin B_6_ levels (fourth quartile (Q4) vs. Q1, OR of 1.676, 95%CI of 1.092 to 2.574, *p* = 0.018, *p* for trend of 0.030). Stratified analyses showed that the positive association between serum vitamin B_6_ and lymph node metastasis appeared to be stronger in females, current smokers, current drinkers, and those with a family history of cancer, squamous cell carcinoma, a tumor of 1–3 cm in diameter, or a solitary tumor. Even though serum vitamin B_6_ levels were associated with preoperative NSCLC upstaging, B_6_ did not qualify as a useful biomarker due to weak association and wide confidence intervals. Thus, it would be appropriate to prospectively investigate the relationship between serum vitamin B_6_ levels and lung cancer further.

## 1. Introduction

Lung cancer is one of the most prevalent malignancies and remains the leading cause of cancer death worldwide [1]. Non-small cell lung cancer (NSCLC) accounts for approximately 85% of lung cancers and represents a significant economic and social burden [2,3]. Tumor–nodes–metastasis (TNM) staging is commonly used to clarify the staging of NSCLC and plays an important role in the treatment and prognosis [4]. However, it is difficult to accurately assess tumor staging preoperatively, as the results are mainly obtained by computed tomography, positron emission tomography, and local biopsy, and they are often incomplete and differ from the final pathological results [4,5,6]. This is mainly due to the challenge of carrying out a comprehensive and accurate preoperative assessment of lymph nodes and localized pleural metastases [7,8,9,10,11].

The B vitamins (including vitamin B_1_, B_2_, B_6_, B_9_, and B_12_) play important roles as cofactors in various biological processes, such as tissue metabolism, cellular stress responses, signal transduction pathways, and gene expression regulation [12,13,14]. The relationship between B vitamin levels and the development and progression of lung cancer has been a popular topic, but the results remain inconclusive [15,16,17,18,19,20,21,22]. B vitamins were reported as tumor promoters [15,23,24,25,26], tumor suppressors [19], or being unrelated to carcinogenesis [27,28] in various studies. Meanwhile, there have been few reports on the association of B vitamins with different stages of lung cancer. The present study aimed to investigate the association between B vitamins and intrapulmonary lymph nodes as well as localized pleural metastases in NSCLC patients, and to clarify the association in different populations by stratified analysis.

## 2. Materials and Methods

### 2.1. Study Population

This retrospective study was approved by the Ethics Committee of Chinese PLA General Hospital (No. S2022-615-01) and was conducted in accordance with the Declaration of Helsinki (as revised in 2013). The study was registered in the Chinese Clinical Trial Registry (chictr.org.cn, ChiCTR2300069309). Informed consent was waived for this retrospective analysis. From January 2016 to December 2018, patients who underwent lung surgery in our hospital for suspected NSCLC were screened for inclusion. The inclusion criteria were as follows: (1) age ≥ eighteen years old; (2) preoperatively evaluated for the absence of distant metastasis; (3) lung nodule(s) or mass(es) treated by surgical resection for suspected NSCLC; (4) no chemotherapy, radiotherapy, targeted drug therapy, or immunotherapy prior to surgery; (5) received serum B vitamin testing (fasting for more than eight hours) within three days before surgery; (6) the diets of patients were arranged by the Nutrition Department, and the daily vitamin B_6_ intake was about 2 mg, which met the Chinese Daily Reference Intake (DRI) recommendations and ensured the uniformity of dietary intake; and (7) no nutritional support therapy or additional nutritional supplements in the past year. The exclusion criteria included: (1) nutritional status score ≥ 1 or total score ≥ 3 in the Nutrition Risk Screening 2002; (2) comorbidities such as uncontrolled diabetes, autoimmune disease, extrapulmonary malignancy, or severe renal or hepatic insufficiency; (3) pregnant female patients during; (4) taking antiepileptics, non-steroidal anti-inflammatory drugs, or oral contraceptives within six months; and/or (5) meeting conditions that the investigator believed may influence vitamin B metabolism. In total, 1498 patients were enrolled in this study.

### 2.2. Data Collection

Clinical data were collected using the medical record system of our institute, and included age, sex, body mass index (BMI), family history of cancer, marital status, smoking history, alcohol consumption, the season of blood sampling for vitamin testing, educational level, surgical records, pathological findings including diameter and number of tumor(s), lung computed tomography report for the density of tumor(s), and serum levels of vitamins B_1_, B_2_, B_6_, B_9_, and B_12_.

### 2.3. Serum Vitamin Level Testing

We used a vitamin analyzer approved by the Chinese National Medical Products Administration (LK3000V, Tianjin Lanbiao Electronic Technology Development Co., Ltd., Tianjin, China) to detect the serum content of vitamin B_1_, vitamin B_2_, vitamin B_6_, vitamin B_9_, and vitamin B_12_ in the form of thionine, riboflavin, pyridoxal 5′ phosphate (PLP), L-5,6,7,8-tetrahydrofolic acid, and cobalamin, respectively. The analyzer enabled the use of an electrochemiluminescence method to deposit the measured substance on the sensor surface; then, the reverse voltage was applied to dissolve the substance accumulated on the sensor and the content was analyzed based on the polarization curve of the dissolution process. The normal thresholds were as follows: vitamin B_1_, 50–150 nmol/L, vitamin B_2_, 4.26–18.42 mg/L; vitamin B_6_, 14.6–72.9 nmol/L; vitamin B_9_, 6.8–36.3 nmol/L; and vitamin B_12_, 200–900 pg/mL.

### 2.4. Intrapulmonary Metastasis Status Classification

We evaluated intrapulmonary lymph nodes and pleural metastases using surgical records and pathology reports. The location and number of metastases were recorded. All the metastatic findings were pathologically confirmed according to the World Health Organization Classification of Thoracic Tumors 2021 [29] and the 8th edition of the Pathological Tumor–Node–Metastasis (pTNM) Staging from the Union for International Cancer Control [4]. Patients were then divided into two groups based on the presence or absence of intrapulmonary metastases.

### 2.5. Covariates

Based on previous experience and studies [18,23,27,30], clinical characteristics were evaluated as potential confounders of the association between serum vitamin levels and intrapulmonary metastasis status, including age, sex, BMI, family history of cancer, smoking history, alcohol consumption, the season of blood collection for vitamin testing, educational level, and tumor type (pathology, diameter, number, and density).

### 2.6. Statistical Analyses

Continuous variables are presented as mean ± standard deviation (normal distribution) or median [interquartile range] (non-normal distribution). Categorical variables are presented as numbers and percentages. Quantitative variables were compared between groups using Student’s *t*-test, analysis of variance, or non-parametric tests, while comparisons of categorical variables between groups were made using chi-squared tests.

Univariate and multivariate logistic regression analyses were used to evaluate the associations between serum vitamin levels and the presence of lung cancer or intrapulmonary metastases. Patients were then divided into four groups by quartiles of the serum vitamins selected by multivariate logistic regression analysis. A stratified analysis was used to examine the vitamin–metastasis association for different levels of factors including: age (<40, 40–60, or >60 years), sex, BMI (<18.5, 18.5–24, or >24 kg/m^2^), family history of tumors (yes or no), smoking history (never a smoker, ex-smoker, or current smoker), alcohol consumption (yes or no), and the pathology (adenocarcinoma or squamous cell carcinoma), diameter (<1, 1–3, or >3 cm), number (solitary or multiple), and density (solid or subsolid) of the tumor(s). Estimated effects were reported as odds ratios (OR) and 95% confidence intervals (95% CI). The last three quartiles of serum vitamins were each compared to the first quartile with the lowest concentration. Tests for linear trends across serum vitamin quartiles were performed by assigning medians to each quartile and calculating coefficients for the quartile variables. *p*-values for interactions were also assessed by likelihood ratio tests comparing regression models with and without the cross-product terms for each assessed factor and serum vitamin levels (quartiles).

All analyses were performed in R software (version 4.2.2, The Free Software Foundation, Boston, MA, USA) and IBM SPSS Statistics for Windows, version 26.0 (IBM Corp., Armonk, NY, USA). A two-sided *p*-value < 0.05 was considered statistically significant for all tests.

## 3. Results

### 3.1. Participant Characteristics

A total of 1498 patients with suspected NSCLC who underwent lung surgery were enrolled, of whom 1283 patients had postoperative pathologically confirmed NSCLC and 215 patients had benign pulmonary nodules (Table 1). Compared to patients with benign nodules, NSCLC patients were older (59 years, *p* < 0.001), had more male patients (49.6%, *p* = 0.001), larger nodule diameters (*p* = 0.018), more subsolid nodules (47.4%, *p* < 0.001), and higher vitamin B_6_ levels (35.6 nmol/L vs. 32.4 nmol/L, *p* = 0.023).

In 1283 NSCLC patients, 276 patients had intrapulmonary metastases, including 223 (80.8%) patients with lymph node metastases (metastatic group) and 53 (19.2%) patients with localized pleural metastases (non-metastatic group) (Table 2). Age, BMI, marital status, and the season of blood sampling were similar in both groups. Compared to the non-metastatic group, the metastatic group had more female patients (62.3%, *p* < 0.001), more patients with no family history of cancer (83.7%, *p* = 0.044), more current smokers (32.6%, *p* < 0.001) and current drinkers (35.5%, *p* = 0.014), more squamous cell carcinoma (21.0%, *p* < 0.001), a larger tumor size (2.5 [1.8, 3.5] cm vs. 1.5 [1.0, 2.2] cm, *p* < 0.001), more multiple tumors (19.9%, *p* < 0.001), and more solid tumors (79.3%, *p* < 0.001). For serum vitamins, the metastatic group had a higher vitamin B_6_ level (36.7 nmol/L vs. 35.1 nmol/L, *p* = 0.037) and a lower vitamin B_12_ level (*p* = 0.039) than the non-metastatic group. There were no significant differences in the serum vitamin B_1_, B_2_, and B_9_ levels between the two groups (Table 2).

### 3.2. Association of Serum B Vitamin Levels with Presence of Lung Cancer

None of the serum B vitamins showed a significant association with the presence of lung cancer in the univariate logistic regression (Table 3). In multivariate analysis, age and a subsolid tumor were positively associated with the presence of lung cancer.

### 3.3. Association of Serum B Vitamin Levels with Intrapulmonary Metastases

Among the serum B vitamins, only vitamin B_6_ levels showed a positive association with intrapulmonary metastases in a multivariate logistic regression (OR: 1.016; 95%CI: 1.002–1.031) (Table 4). Other significantly associated factors included: female gender, the diameter and number of the tumors as risk factors, and the density of the tumor as a protective factor. Meanwhile, there were no significant differences in the demographic, clinical, or tumor characteristics of patients in different quartiles for serum vitamin B_6_, except for those with different smoking histories (*p* < 0.032) (Table 5).

After multivariable adjustment, the highest quartile (Q4) of serum vitamin B_6_ was significantly associated with a 67.6% higher risk of metastasis compared with those in the lowest quartile (Q1) (OR = 1.676, 95% CI = 1.092–2.574, *p* = 0.018), with a *p* for trend of 0.030 (Table 6). We also evaluated the effect modification of the association between serum vitamin B_6_ and metastases by age, sex, BMI, family history of cancer, smoking history, alcohol consumption, and the pathology, diameter, number, and density of the tumor(s) (Table 6). The risk association appeared stronger for women (Q4 vs. Q1: OR of 1.968, 95% CI of 1.144 to 3.386, *p* = 0.014, *p* for trend = 0.014). Patients with a family history of cancer also appeared to have experienced a stronger association between vitamin B_6_ and metastasis (Q4 vs. Q1: OR of 5.337, 95% CI of 1.492 to 19.093, *p* = 0.010, *p* for trend = 0.010). The association between serum vitamin B_6_ and intrapulmonary metastasis also appeared to be stronger for current smokers, current drinkers, and patients with squamous cell carcinoma, a tumor of 1–3 cm in diameter, or a solitary tumor (Table 6). Meanwhile, age, sex, smoking history, alcohol consumption, and tumor type (pathology, diameter, number, and density) showed significant interactions with vitamin B_6_.

## 4. Discussion

In this retrospective study, we found that serum vitamin B_6_ levels might be positively associated with intrapulmonary lymph nodes and/or localized pleural metastases in NSCLC patients, and the risk association was stronger for women, current smokers, current drinkers, and patients with squamous cell carcinoma, tumors 1–3 cm in diameter, or a solitary tumor. However, even though serum vitamin B_6_ levels were associated with preoperative NSCLC upstaging, B_6_ did not qualify as a useful biomarker due to weak associations and wide confidence intervals. Preoperative vitamin B_6_ levels might have some value in the preoperative evaluation of intrapulmonary lymph nodes and/or localized pleural metastases in NSCLC; thus, it would be appropriate to prospectively investigate the relationship between serum vitamin B_6_ levels and lung cancer further.

Vitamin B_6_ promotes cell growth, differentiation, proliferation, and metastasis [31] and has adjuvant anti-inflammatory and antioxidant effects [32]. Although large cohort studies have been conducted, the association between vitamin B_6_ intake, blood levels, and catabolic levels with lung cancer remains inconclusive. Vitamin B_6_ has been implicated as a tumor promoter [15,23,24,25,26], tumor suppressor [19], or being unrelated to carcinogenesis [27,28] in various studies. This contradiction may be attributed to the significant variation in vitamin B_6_ among populations of different races, diets, lifestyles, and vitamin supplementation habits [33,34]. In our study, we found that patients with lung cancer had higher serum vitamin B_6_ levels than patients with benign lung nodules, while patients with intrapulmonary metastases had higher serum vitamin B_6_ levels than non-metastatic patients, i.e., serum vitamin B_6_ levels increased with increasing tumor status. Theoretically, vitamin B_6_ could promote tumor cell growth, differentiation, and proliferation, as well as be a risk factor for lung carcinogenesis [35,36]. On the other hand, it could act as an antitumor factor with regard to the inflammatory response caused by tumor cells [37,38,39]. Several studies have shown that vitamin B_6_ can act as a promotor in tumors by affecting DNA stability [40,41] and the activation of antioxidant enzymes [42,43,44,45], which are involved in the regulation of post-translational modifications of activated proteins. The relationship of vitamin B_6_ with post-translational modifications of key proteins such as NF-κB [46,47], as well as inflammasomes such as NLRP3 [48,49] and receptor-interacting protein 140 (RIP140) [47,50], has received significant attention. Although there are no specific findings related to the mechanism of vitamin B_6_ and lung cancer metastasis, we speculated that these two aspects mentioned above together determine the effect of vitamin B_6_ on tumors. Factors such as demographics and tumor type may play different roles in both aspects, leading to different conclusions. In our study, the positive relationship between vitamin B_6_ and intrapulmonary metastasis suggests that vitamin B_6_ may contribute to tumor metastasis in NSCLC patients.

We measured serum PLP as an assessment of vitamin B_6_ levels. Vitamin B_6_ consists of pyridoxal (PN), pyridoxamine (PM), pyridoxal (PL), and their phosphorylated derivatives pyridoxal 5’-phosphate (PNP), pyridoxamine 5’-phosphate (PMP), and PLP [51]. PLP is the bioactive form of vitamin B_6_ in vivo and has been the common measure of vitamin B_6_ status testing in previous studies [19,38,51,52,53,54]. However, PLP is considered unstable because it may be influenced by inflammatory status, alkaline phosphatase levels, serum albumin, and inorganic phosphate [55]. Recent studies have used plasma vitamer ratios (PAr), calculated as PA:(PLP + PL), to eliminate the influence of the above factors [55,56]. However, PAr requires the simultaneous measurement of three indices, and mainly reflects the catabolism of vitamin B_6_ in the inflammatory state. In contrast, PLP serves as a representation of the true vitamin B_6_ levels and can directly reflect the relationship between vitamin B_6_ and lung cancer. PLP is also more compatible with clinical applications because it requires less blood sampling and is less expensive.

A stratified analysis was performed to clarify the association of vitamin B_6_ with intrapulmonary metastasis in different populations. We found that the risk association was much stronger in some situations. Vitamin B_6_ served as a strong risk factor in females, current smokers, current drinkers, and those with a family history of cancer. The study by Fanidi et al. also found a positive trend for elevated PLP levels and lung cancer risk in their cohort of Asian women [19]. In addition, an elevated serum vitamin B_6_ level, even within the normal range, was a risk factor for those with a family history of cancer, suggesting a possible genetic link might exist between them. Meanwhile, smoking and alcohol consumption are well-known risk factors for lung cancer [15,19]. Vitamin B_6_ may be associated with smoking-induced inflammation and immune activation, which was also one of the mechanisms related to smoking in lung cancer development [30,57,58].

Interestingly, pathology, tumor size, tumor number, and morphology all seemed to influence the strength of vitamin B_6_ as a risk factor for intrapulmonary metastasis. Previous studies showed that increased vitamin B_6_ catabolism was positively associated with the risk of lung carcinogenesis, especially in squamous cell carcinoma [15]. This finding was confirmed in our study. In addition, we found a stronger risk association in patients with a solitary tumor or a tumor 1–3 cm in diameter. A consistent trend toward an increased risk of metastasis with increasing vitamin B_6_ levels was also found in the subsolid tumor population. These associations have not been previously studied, and the theoretical basis for them remains unclear. In clinical practice, patients with a solitary, subsolid tumor or a tumor 1–3 cm in diameter without metastases are good candidates for surgery. If the vitamin–metastasis association could be confirmed by future studies, vitamin B_6_ could be of great value in supporting surgical treatment as a preoperative predictor for metastasis assessment.

In this study, we found that age, sex, smoking history, alcohol consumption, and several characteristics of tumors had significant interactions with vitamin B_6_, respectively. That is, different degrees of the above factors could significantly affect the strength of the association between vitamin B_6_ and intrapulmonary metastasis. The results of the interaction and stratified analysis further confirmed that women, current smokers, current drinkers, patients with squamous cell carcinoma, those with tumors 1–3 cm in size, and those with a solitary tumor may be a part of the special population whose vitamin B_6_ levels should be of great concern. In addition, although there was a significant interaction between age and vitamin B_6_, the *p* for trend was found to be statistically insignificant (ranging from 0.112 to 0.119), which might be caused by the sample size. Furthermore, there was no significant interaction between family history and vitamin B_6_, suggesting that the effect of family history on the association should be further investigated.

Tumor staging is crucial in cancer treatment and determines the outcome. The pTNM staging results could not be obtained comprehensively before surgery. Clinical TNM staging might be relatively accurate in evaluating the tumor stage, but are not precise in the evaluation of the nodule and metastasis stage, especially in the early stage of lung cancer. In our study, we found 53 patients (53/1283) who underwent surgery had postoperative confirmation of localized pleural metastases, suggesting that surgical treatment may not be appropriate for them. Vitamin B_6_ testing may have the potential to improve preoperative TNM staging. Further studies with larger sample sizes are needed to verify the value of vitamin B_6_ in surgical management.

Notably, the results obtained in this study should be treated with caution. On one hand, the association between lung cancer and vitamins is very complex. Many factors may affect serum vitamin levels, including dietary habits, vitamin supplements, body metabolism, and a variety of complications. In this study, we collected as much nutritional information as possible and excluded conditions that could affect serum vitamins. However, more rigorous prospective studies are necessary to properly research the pathogenetic relationship between vitamin levels in the blood and the occurrence and progression of lung cancer. On the other hand, even though vitamin B_6_ levels showed positive association with intrapulmonary metastases in NSCLC patients, the relationship was weak, and the confidence intervals overlapped hugely. Thus, the statistically significant difference between groups of patients could not be translated into a predictor on the level of individual patients based on the results of this study. Vitamin B_6_ levels might have some value in the preoperative evaluation of intrapulmonary metastases in NSCLC, but more research is needed before conclusions can be drawn.

This study had several limitations. First, the sample size of this study was limited. Studies with larger sample sizes are needed for further validation. Second, there may be an interaction between vitamin B_6_ and tumor metastasis status. The study identified a positive association between vitamin B_6_ and intrapulmonary metastases, but the cause-and-effect relationship between them was not clear. Third, this study was retrospective; therefore, prospective cohort studies are needed to explore the mechanism of action of vitamin B_6_ levels and lung cancer. Fourth, we collected as much information as possible about patients’ dietary intake, nutritional status, and nutritional supplementation, but as a retrospective study, nutrition-related clinical information was not sufficiently detailed and might influence the results.

## 5. Conclusions

In conclusion, our study demonstrated a possible positive association between serum vitamin B_6_ levels and intrapulmonary lymph nodes and/or localized pleural metastases in NSCLC patients, and the association was stronger in specific populations including women, current smokers, current drinkers, patients with squamous cell carcinoma, those with tumors 1–3 cm in diameter, and those with a solitary tumor. However, B_6_ did not qualify as a useful biomarker due to weak associations and wide confidence intervals. Preoperative vitamin B_6_ levels might have some value in the preoperative evaluation of intrapulmonary lymph nodes and/or localized pleural metastases in NSCLC, and thus the relationship between serum vitamin B_6_ levels and lung cancer should be prospectively investigated in the future.

## Figures and Tables

**Table 1:** Basic characteristics of all patients enrolled in this study.

Characteristics	All Patients (n = 1498)	Patients with Non-Small Cell Lung Cancer	Patients with Benign Lung Nodule	*p*-Value
		(n = 1283)	(n = 215)	
Age (year), median [IQR]	58 [53, 64]	59 [51, 65]	52 [48, 55]	<0.001 ^†^
Sex				
Male, N (%)	717 (47.9)	636 (49.6)	81 (37.7)	0.001 ^‡^
Female, N (%)	781 (52.1)	647 (50.4)	134 (62.3)	
BMI (kg/m^2^), median [IQR]	24.4 [22.3, 26.8]	24.4 [22.5, 26.6]	24.9 [22.7, 27.3]	0.323 ^†^
Family history of cancer				
No, N (%)	1188 (79.3)	1018 (79.3)	170 (79.1)	0.926 ^‡^
Yes, N (%)	310 (20.7)	265 (20.7)	45 (20.9)	
Marital status				
Married, N (%)	1466 (97.9)	1256 (97.9)	210 (97.7)	0.514 ^‡^
Never married, N (%)	8 (0.5)	6 (0.5)	2 (0.9)	
Divorced or widowed, N (%)	24 (1.6)	21 (1.6)	3 (1.4)	
Smoking history				
Never a smoker, N (%)	937 (62.6)	807 (62.9)	130 (60.5)	0.308 ^‡^
Ex-smoker, N (%)	207 (13.8)	182 (14.2)	25 (11.6)	
Current smoker, N (%)	354 (23.6)	294 (22.9)	60 (27.9)	
Alcohol consumption				
No, N (%)	1042 (69.6)	904 (70.5)	138 (64.2)	0.064 ^‡^
Yes, N (%)	456 (30.4)	379 (29.5)	77 (35.8)	
Season of blood sampling for vitamin testing				
June–September, N (%)	616 (41.1)	529 (41.2)	87 (40.5)	0.833 ^‡^
Other, N (%)	882 (58.9)	754 (58.8)	128 (59.5)	
Educational level				
No greater than elementary school, N (%)	203 (13.5)	179 (14.0)	24 (11.2)	0.985 ^‡^
High school graduation, N (%)	693 (46.3)	585 (45.6)	108 (50.2)	
University or postgraduate graduation, N (%)	602 (40.2)	519 (40.5)	83 (38.6)	
Diameter of tumor (cm), median [IQR]	1.5 [1.0, 2.4]	1.6 [1.0, 2.5]	1.5 [0.9, 2.4]	0.018 ^†^
Number of tumor(s)				
Solitary, N (%)	1405 (93.8)	1197 (93.3)	208 (96.7)	0.053 ^‡^
Multiple, N (%)	93 (6.2)	86 (6.7)	7 (3.3)	
Density of tumor				
Solid, N (%)	854 (57.0)	675 (52.6)	179 (83.3)	<0.001 ^‡^
Subsolid, N (%)	644 (43.0)	608 (47.4)	3 6(16.7)	
Serum vitamin levels				
Vitamin B_1_, nmol/L	88.3 [75.2, 108.2]	88.3 [77.9, 104.1]	88.8 [73.5, 108.6]	0.685 ^†^
Vitamin B_2_, mg/L	4.5 [4.0, 5.1]	4.5 [4.1, 5.1]	4.6 [4.1, 5.3]	0.538 ^†^
Vitamin B_6_, nmol/L	35.1 [29.3, 39.8]	35.6 [30.2, 40.4]	32.4 [25.8, 38.5]	0.023 ^†^
Vitamin B_9_, nmol/L	20.4 [16.6, 24.5]	20.4 [17.4, 24.0]	20.8 [17.5, 24.2]	0.643 ^†^
Vitamin B_12_, pg/mL	432.4 [348.2, 519.5]	434.8 [360.4, 529.3]	423.7 [326.6, 537.2]	0.688 ^†^

IQR, interquartile range; BMI, body mass index. ^†^ *p*-value based on non-parametric test (continuous variable). ^‡^ *p*-value based on chi-squared test (categorical variable).

**Table 2:** Basic characteristics of NSCLC patients in this study.

Characteristics	Total (n = 1283)	Patients with Intrapulmonary Metastases	Patients without Intrapulmonary Metastases	*p*-Value
		(n = 276)	(n = 1007)	
Age (year), median [IQR]	59 [51, 65]	59 [52, 65]	59 [51, 65]	0.800 ^†^
Sex				
Male, N (%)	636 (49.6)	104 (37.7)	532 (52.8)	<0.001 ^‡^
Female, N (%)	647 (50.4)	172 (62.3)	475 (47.2)	
BMI (kg/m^2^), median [IQR]	24.4 [22.5, 26.6]	24.4 [22.1, 26.9]	24.4 [22.5, 26.5]	0.909 ^†^
Family history of cancer				
No, N (%)	1018 (79.3)	231 (83.7)	787 (78.2)	0.044 ^‡^
Yes, N (%)	265 (20.7)	45 (16.3)	220 (21.8)	
Marital status				
Married, N (%)	1256 (97.9)	270 (97.8)	985 (97.9)	0.919 ^‡^
Never married, N (%)	6 (0.5)	1 (0.4)	5 (0.5)	
Divorced or widowed, N (%)	21 (1.6)	5 (1.8)	16 (1.6)	
Smoking history				
Never a smoker, N (%)	807 (62.9)	134 (48.6)	673 (66.8)	<0.001 ^‡^
Ex-smoker, N (%)	182 (14.2)	52 (18.8)	130 (12.9)	
Current smoker, N (%)	294 (22.9)	90 (32.6)	204 (20.3)	
Alcohol consumption				
No, N (%)	904 (70.5)	178 (64.5)	726 (72.1)	0.014 ^‡^
Yes, N (%)	379 (29.5)	98 (35.5)	281 (27.9)	
Season of blood sampling for vitamin testing				
June–September, N (%)	529 (41.2)	126 (45.7)	403 (40.0)	0.092 ^‡^
Other, N (%)	754 (58.8)	150 (54.3)	604 (60.0)	
Educational level				
No greater than elementary school, N (%)	179 (14.0)	42 (15.2)	137 (13.6)	0.007 ^‡^
High school graduation, N (%)	585 (45.6)	145 (52.5)	440 (43.7)	
University or postgraduate graduation, N (%)	519 (40.5)	89 (32.2)	430 (42.7)	
Pathology of tumor				
Adenocarcinoma, N (%)	1125 (87.7)	218 (79.0)	907 (90.1)	<0.001 ^‡^
Squamous cell carcinoma, N (%)	158 (12.3)	58 (21.0)	100 (9.9)	
Intrapulmonary metastasis				
Lymph node, N (%)	223 (17.5)	223 (80.8)		NA ^§^
Localized pleura, N (%)	53 (4.1)	53 (19.2)		
None, N (%)	1007 (78.4)		1007 (100)	
Diameter of tumor (cm), median [IQR]	1.6 [1.0, 2.5]	2.5 [1.8, 3.5]	1.5 [1.0, 2.2]	<0.001 ^†^
Number of tumor(s)				
Solitary, N (%)	1197 (93.3)	221 (80.1)	976 (96.9)	<0.001 ^‡^
Multiple, N (%)	86 (6.7)	55 (19.9)	31 (3.1)	
Density of tumor				
Solid, N (%)	675 (52.6)	219 (79.3)	456 (45.3)	<0.001 ^‡^
Subsolid, N (%)	608 (47.4)	57 (20.7)	551 (54.7)	
Serum vitamin levels				
Vitamin B_1_, nmol/L	88.3 [77.9, 104.1]	89.3 [77.7, 108.6]	88.1 [78.0, 102.7]	0.237 ^†^
Vitamin B_2_, mg/L	4.5 [4.1, 5.1]	4.6 [4.2, 5.2]	4.5 [4.1, 5.1]	0.162 ^†^
Vitamin B_6_, nmol/L	35.6 [30.2, 40.4]	36.7 [30.5, 42.0]	35.1 [30.2, 40.2]	0.037 ^†^
Vitamin B_9_, nmol/L	20.4 [17.4, 24.0]	20.2 [17.1, 24.5]	20.5 [17.5, 23.9]	0.929 ^†^
Vitamin B_12_, pg/mL	434.8 [360.4, 529.3]	418.2 [340.6, 532.3]	437.8 [365.7, 527.4]	0.039 ^†^

IQR, interquartile range; BMI, body mass index. ^†^ *p*-value based on non-parametric test (continuous variable). ^‡^ *p*-value based on chi-squared test (categorical variable). ^§^ used for grouping, not for comparison between groups.

**Table 3:** Association between clinical characteristics and presence of lung cancer in all patients.

Characteristics	Univariate Analysis		Multivariate Analysis	
	OR (95% CI)	*p*-Value	OR (95% CI)	*p*-Value
Age, year (continuous)	1.060 (1.044, 1.076)	<0.001	1.073 (1.056, 1.090)	<0.001
Female (vs. male)	0.615 (0.457, 0.827)	0.001		
BMI, kg/m^2^ (continuous)	0.976 (0.931, 1.023)	0.305		
Family history of cancer (vs. none)	0.983 (0.689, 1.403)	0.926		
Married (vs. other)	1.419 (0.499, 4.041)	0.512		
Current smoker (vs. never a smoker and ex-smoker)	0.903 (0.764, 1.068)	0.232		
Current drinker (vs. not)	0.751 (0.555, 1.018)	0.065		
Blood sampling in Jun–Sep (vs. other)	0.969 (0.722, 1.300)	0.833		
High educational level (vs. low)	0.980 (0.793, 1.211)	0.852		
Diameter of tumor, cm (continuous)	1.086 (0.973, 1.212)	0.143		
Multiple tumors (vs. solitary tumor)	2.135 (0.974, 4.677)	0.058		
Subsolid tumor (vs. solid tumor)	4.479 (3.080, 6.513)	<0.001	5.690 (3.850, 8.408)	<0.001
Serum vitamin levels				
Vitamin B_1_ (continuous)	0.999 (0.992, 1.005)	0.728		
Vitamin B_2_ (continuous)	0.985 (0.911, 1.065)	0.711		
Vitamin B_6_ (continuous)	1.009 (0.944, 1.024)	0.251		
Vitamin B_9_ (continuous)	0.995 (0.968, 1.023)	0.731		
Vitamin B_12_ (continuous)	1.000 (0.999, 1.001)	0.759		

OR, odds ratio; 95% CI, 95% confidence interval; BMI: body mass index.

**Table 4:** Association between clinical characteristics and presence of intrapulmonary metastases in NSCLC patients.

Characteristics	Univariate Analysis		Multivariate Analysis	
	OR (95% CI)	*p*-Value	OR (95% CI)	*p*-Value
Age, year (continuous)	1.003 (0.989, 1.017)	0.703		
Female (vs. male)	1.852 (1.410, 2.434)	<0.001	1.439 (1.039, 1.993)	0.028
BMI, kg/m^2^ (continuous)	1.006 (0.963, 1.051)	0.786		
Family history of cancer (vs. none)	0.697 (0.490, 0.991)	0.045		
Married (vs. other)	1.181 (0.481, 2.903)	0.716		
Current smoker (vs. never a smoker and ex-smoker)	1.511 (1.298, 1.759)	<0.001		
Current drinker (vs. not)	1.422 (1.073, 1.887)	0.014		
Blood sampling in Jun–Sep (vs. other)	0.794 (0.607, 1.039)	0.093		
High educational level (vs. low)	0.778 (0.643, 0.942)	0.010		
Squamous cell carcinoma (vs. adenocarcinoma)	2.413 (1.691, 3.444)	<0.001		
Diameter of tumor, cm (continuous)	1.686 (1.530, 1.859)	<0.001	1.495 (1.337, 1.672)	<0.001
Multiple tumors (vs. solitary tumor)	7.835 (4.928, 12.459)	<0.001	26.004 (14.517, 46.580)	<0.001
Subsolid tumor (vs. solid tumor)	0.215 (0.157, 0.296)	<0.001	0.220 (0.142, 0.342)	<0.001
Serum vitamin levels				
Vitamin B_1_ (continuous)	1.004 (0.999, 1.010)	0.134		
Vitamin B_2_ (continuous)	1.013 (0.943, 1.088)	0.725		
Vitamin B_6_ (continuous)	1.016 (1.004, 1.028)	0.010	1.016 (1.002, 1.031)	0.021
Vitamin B_9_ (continuous)	1.004 (0.979, 1.030)	0.729		
Vitamin B_12_ (continuous)	0.999 (0.998, 1.000)	0.074		

OR, odds ratio; 95% CI, 95% confidence interval; BMI: body mass index.

**Table 5:** Characteristics of NSCLC patients by quartile of serum vitamin B_6_ in this study.

Characteristics	Serum Vitamin B_6_ Quartile				*p*-Value
	Q1	Q2	Q3	Q4	
	(n = 322)	(n = 320)	(n = 321)	(n = 320)	
Age (year), median [IQR]	59 [51, 64]	59 [51, 66]	58 [50, 64]	59 [53, 66]	0.369 ^†^
Sex					
Male, N (%)	162 (50.3)	165 (51.6)	158 (49.2)	151 (47.2)	0.724 ^‡^
Female, N (%)	160 (49.7)	155 (48.4)	163 (50.8)	169 (52.8)	
BMI (kg/m^2^), median [IQR]	24.5 [22.2, 26.5]	24.5 [22.6, 26.6]	24.3 [22.7, 26.5]	24.3 [22.3, 26.6]	0.835 ^†^
Family history of cancer					
No, N (%)	245 (76.1)	254 (79.4)	257 (80.1)	262 (81.9)	0.329 ^‡^
Yes, N (%)	77 (23.9)	66 (20.6)	64 (19.9)	58 (18.1)	
Smoking history					
Never a smoker, N (%)	198 (61.5)	207 (64.7)	209 (65.1)	193 (60.3)	0.032 ^‡^
Ex-smoker, N (%)	47 (14.6)	39 (12.2)	33 (10.3)	63 (19.7)	
Current smoker, N (%)	77 (23.9)	74 (23.1)	79 (24.6)	64 (20.0)	
Alcohol consumption					
No, N (%)	228 (70.8)	222 (69.4)	242 (75.4)	212 (66.3)	0.083 ^‡^
Yes, N (%)	94 (29.2)	98 (30.6)	79 (24.6)	108 (33.8)	
Pathology of tumor					
Adenocarcinoma, N (%)	282 (87.6)	282 (88.1)	285 (88.8)	276 (86.3)	0.794 ^‡^
Squamous cell carcinoma, N (%)	40 (12.4)	38 (11.9)	36 (11.2)	44 (13.8)	
Diameter of tumor (cm), median [IQR]	1.6 [1.0, 2.5]	1.5 [1.0, 2.5]	1.5 [1.0, 2.5]	1.8 [1.0, 2.7]	0.277 ^†^
Number of tumor(s)					
Solitary, N (%)	297 (92.2)	305 (95.3)	304 (94.7)	291 (90.9)	0.089 ^‡^
Multiple, N (%)	25 (7.8)	15 (4.7)	17 (5.3)	29 (9.1)	
Density of the tumor					
Solid, N (%)	176 (54.7)	166 (51.9)	166 (51.7)	167 (52.2)	0.864 ^‡^
Subsolid, N (%)	146 (45.3)	154 (48.1)	155 (48.3)	153 (47.8)	

IQR, interquartile range; BMI, body mass index; Q, quartile of vitamin B_6_. ^†^ *p*-value based on non-parametric test (continuous variable). ^‡^ *p*-value based on chi-squared test (categorical variable).

**Table 6:** Multivariable-adjusted odds ratios and 95% confidence intervals of intrapulmonary metastases by quartile of serum vitamin B_6_, stratified by age, sex, BMI, family history of cancer, smoking history, alcohol consumption, tumor pathology and characteristics in NSCLC patients in this study *.

Stratified Characteristics			Vit B_6_ (μmol/L) (in Quartiles)				*p* for Trend ^‡^	*p* for Interaction ^§^
			Q1	Q2	Q3	Q4		
			(n = 322)	(n = 320)	(n = 321)	(n = 320)		
All patients	(n = 1283)	OR	1.0 (Ref)	1.208 (0.775, 1.885)	1.123 (0.717, 1.759)	1.676 (1.092, 2.574)	0.030	
		*p*-value ^†^		0.404	0.612	0.018		
Age, y	<40 (n = 43)	OR	1.0 (Ref)	NA ^¶^	NA ^¶^	NA ^¶^	NA ^¶^	0.032
		*p*-value ^†^						
	40 to 60 (n = 693)	OR	1.0 (Ref)	1.432 (0.769, 2.669)	1.130 (0.593, 2.152)	1.803 (0.978, 3.325)	0.112	
		*p*-value ^†^		0.258	0.711	0.059		
	>60 (n = 547)	OR	1.0 (Ref)	1.172 (0.602, 2.283)	1.084 (0.549, 2.137)	1.738 (0.914, 3.305)	0.119	
		*p*-value ^†^		0.640	0.817	0.092		
Sex	Male (n = 636)	OR	1.0 (Ref)	0.966 (0.473, 1.946)	0.731 (0.338, 1.579)	1.172 (0.569, 2.414)	0.838	<0.001
		*p*-value ^†^		0.926	0.425	0.667		
	Female (n = 647)	OR	1.0 (Ref)	1.252 (0.704, 2.229)	1.404 (0.799, 2.466)	1.968 (1.144, 3.386)	0.014	
		*p*-value ^†^		0.444	0.238	0.014		
BMI, kg/m^2^	<18.5 (n = 11)	OR	1.0 (Ref)	NA ^¶^	NA ^¶^	NA ^¶^	NA ^¶^	0.527
		*p*-value ^†^						
	18.5 to 24.0 (n = 553)	OR	1.0 (Ref)	1.478 (0.749, 2.917)	0.949 (0.472, 1.909)	1.947 (0.999, 3.793)	0.137	
		*p*-value ^†^		0.260	0.884	0.050		
	>24.0 (n = 719)	OR	1.0 (Ref)	1.025 (0.560, 1.873)	1.245 (0.683, 2.271)	1.633 (0.920, 2.898)	0.069	
		*p*-value ^†^		0.937	0.474	0.094		
Family history of cancer	No (n = 1018)	OR	1.0 (Ref)	0.997 (0.614, 1.618)	0.847 (0.515, 1.393)	1.375 (0.865, 2.185)	0.252	0.846
		*p*-value †		0.989	0.513	0.179		
	Yes (n = 265)	OR	1.0 (Ref)	4.176 (1.151, 15.146)	4.646 (1.377, 15.674)	5.337 (1.492, 19.093)	0.010	
		*p*-value ^†^		0.030	0.013	0.010		
Smoking history	Never a smoker (n = 807)	OR	1.0 (Ref)	1.428 (0.754, 2.704)	1.257 (0.653, 2.421)	1.356 (0.709, 2.593)	0.476	<0.001
		*p*-value ^†^		0.274	0.494	0.357		
	Ex-smoker (n = 182)	OR	1.0 (Ref)	1.684 (0.564, 5.026)	0.528 (0.138, 2.022)	1.810 (0.694, 4.722)	0.393	
		*p*-value ^†^		0.350	0.351	0.225		
	Current smoker (n = 294)	OR	1.0 (Ref)	0.697 (0.298, 1.630)	1.211 (0.555, 2.642)	2.462 (1.104, 5.491)	0.016	
		*p*-value ^†^		0.405	0.631	0.028		
Alcohol consumption	Never a drinker (n = 904)	OR	1.0 (Ref)	1.441 (0.837, 2.483)	0.818 (0.463, 1.445)	1.371 (0.797, 2.358)	0.642	<0.001
		*p*-value ^†^		0.188	0.488	0.254		
	Current drinker (n = 379)	OR	1.0 (Ref)	0.800 (0.357, 1.793)	2.159 (1.008, 4.628)	2.163 (1.041, 4.492)	0.006	
		*p*-value ^†^		0.588	0.048	0.039		
Pathology of tumor	Adenocarcinoma (n = 1125)	OR	1.0 (Ref)	1.124 (0.682, 1.853)	1.193 (0.725, 1.962)	1.360 (0.836, 2.214)	0.211	<0.001
		*p*-value ^†^		0.645	0.487	0.216		
	Squamous cell carcinoma (n = 158)	OR	1.0 (Ref)	1.523 (0.519, 4.470)	0.761 (0.248, 2.333)	3.933 (1.421, 10.888)	0.024	
		*p*-value ^†^		0.444	0.633	0.008		
Diameter of tumor, cm	<1 (n = 370)	OR	1.0 (Ref)	0.339 (0.031, 3.656)	0.450 (0.055, 3.659)	1.069 (0.183, 6.262)	0.898	<0.001
		*p*-value ^†^		0.373	0.455	0.941		
	1–3 (n = 731)	OR	1.0 (Ref)	1.661 (0.944, 2.923)	1.759 (1.000, 3.094)	2.152 (1.233, 3.755)	0.009	
		*p*-value ^†^		0.078	0.050	0.007		
	>3 (n = 182)	OR	1.0 (Ref)	0.684 (0.279, 1.679)	0.455 (0.180, 1.149)	1.406 (0.605, 3.265)	0.645	
		*p*-value ^†^		0.408	0.096	0.429		
Number of tumor(s)	Solitary (n = 1197)	OR	1.0 (Ref)	1.205 (0.752, 1.932)	1.156 (0.719, 1.859)	1.664 (1.051, 2.636)	0.043	<0.001
		*p*-value ^†^		0.439	0.548	0.030		
	Multiple (n = 86)	OR	1.0 (Ref)	1.058 (0.225, 4.985)	0.578 (0.118, 2.826)	1.319 (0.327, 5.323)	0.776	
		*p*-value ^†^		0.943	0.499	0.698		
Density of tumor	Solid (n = 675)	OR	1.0 (Ref)	1.251 (0.760, 2.059)	1.048 (0.634, 1.730)	1.519 (0.932, 2.477)	0.167	<0.001
		*p*-value ^†^		0.379	0.856	0.094		
	Subsolid (n = 608)	OR	1.0 (Ref)	1.113 (0.388, 3.191)	1.628 (0.559, 4.743)	2.468 (0.963, 6.325)	0.038	
		*p*-value ^†^		0.842	0.372	0.060		

OR, odds ratio; Q, quartile of vitamin B_6_; BMI, body mass index. * Adjusted for factors including age, sex, BMI, family history of cancer, smoking history, alcohol consumption, season of blood sampling for serum vitamin testing, educational level, pathology, and tumor type (diameter, number, and density), unless the factor is used for stratification. ^†^ Q2–Q4 of serum vitamin B_6_ are each compared with Q1. ^‡^ *p* for trend is based on the statistical significance of the coefficient of the quartile variable (median value within each quartile). ^§^ *p* for interaction is based on the statistical significance of the cross-product term added to multivariable models. ^¶^ not enough data for stratified analysis.

## Data Availability

The raw data supporting the conclusions of this article will be made available by the authors, without undue reservation.
